# Clear Cell Odontogenic Carcinoma: A Series of Three Cases

**DOI:** 10.3390/dj10030034

**Published:** 2022-02-25

**Authors:** Asad Ullah, Christian Cullen, Samantha N. Mattox, Diana Kozman, Nikhil Patel, Suash Sharma, Rafik Abdelsayed

**Affiliations:** 1 Department of Pathology, Medical College of Georgia, Augusta, GA 30912, USA; aullah@augusta.edu (A.U.); ccullen@augusta.edu (C.C.); smattox@augusta.edu (S.N.M.); npatel4@augusta.edu (N.P.); suash.sharma@va.gov (S.S.); 2 Cleveland Clinic, Cleveland, OH 44195, USA; kozmand@ccf.org; 3 Department of Oral and Maxillofacial Surgery, Dental College of Georgia, Augusta, GA 30912, USA

**Keywords:** odontogenic epithelial neoplasm, radiolucent, dentinoid

## Abstract

Background: Clear cell odontogenic carcinoma (CCOC) is a rare malignant odontogenic epithelial neoplasm of the jaws. It is composed of irregular nests of clear to faintly eosinophilic cells resembling clear cell rests of primitive dental lamina and an intermixed hyalinized fibrous stroma. Most cases occur in the 5th and 6th decades of life, with a female predominance. The mandible is affected more than the maxilla. Clinical features vary from asymptomatic to non-specific pain, ill-defined radiolucency, root resorption, and sometimes soft tissue extension. Histology varies from bland to high grade. CCOC demonstrated a significant tendency to recur. Metastasis typically involves regional lymph nodes, which haves been reported in 20–25% of cases. Pulmonary metastasis rarely occurs. Differential diagnoses are broad and include odontogenic, salivary, melanocytic, and metastatic neoplasia. CCOCs are positive for cytokeratins, mainly AE1/AE3 and CK19. Most cases show EWSR1 rearrangement and rarely, the BRAFV600E mutation. Design: Patient charts were reviewed at our institution. A total of three cases were found in electronic medical records, which were diagnosed as clear cell odontogenic carcinoma over a period of six years (2014–2019). Patient charts were reviewed for medical history and radiology data. The pathology slides were reviewed by one or more faculty members. Results: We present three cases of CCOC, ranging in age from 40 to 69 years (two women and one man). Two cases involved the maxilla and one involved the mandible. Two presented with painful swelling and one with mass recurrence. Radiography results show that two had poorly defined radiolucent lesions, and one was heterogeneous with a small nodule projecting into the maxillary sinus. Histological examination revealed an epithelial neoplasm composed of irregular sheets, cords, and nests of polygonal cells with central hyperchromatic, mildly pleomorphic nuclei surrounded by clear to pale eosinophilic cytoplasm, with occasional mitotic figures. The tumor had infiltrated the bone and soft tissues. Two cases were immunopositive for CK5/6 and one case was positive for p63 and CK19. Interestingly, the eosinophilic dentinoid matrix interspersed among tumor cells in one case was consistent with its odontogenic origin. Histochemical staining showed PAS-positive and diastase-labile intracytoplasmic material consistent with glycogen. Conclusion: Our study highlights the potential diagnostic significance of dentinoid (although reportedly seen in only 7% of cases), along with CK5/6 immunopositivity, in supporting the histologic diagnosis of CCOC among a variety of neoplasia in its differential diagnosis.

## 1. Introduction

Clear cell odontogenic carcinoma (CCOC) is a rare malignant neoplasm of odontogenic epithelium. CCOC most frequently involves the posterior mandible; other common sites include the anterior mandible and maxilla. CCOC commonly occurs in the 6th decade of life with a slight female predilection. Clinically, CCOC presents as jaw expansion, numbness, tooth mobility, and gingival swelling [1]. Radiologically, CCOCs may show unilocular and multilocular radiolucency with poor or well-demarcated borders. Histologically, CCOC exhibit three distinct patterns: monophasic, biphasic, and ameloblastic. The biphasic is by far the most common pattern. Pankeratin and EMA are usually positive in CCOC [2]. DNA microarray analysis is a useful tool for the diagnosis of CCOCs. Approximately 84% of CCOCs express EWSR1-ATF1 rearrangement. Other microscopically similar lesions such as clear cell carcinoma of the salivary gland express the EWSR-ARF1 gene arrangement [3]. Therefore, the diagnosis of CCOC is usually challenging and requires clinical, radiological, and pathological correlations. Treatment options include curettage, wide resection with clear margins, and radiotherapy for soft tissue involvement. The recurrence rate is approximately 87% in patients treated with curettage and as low as 41% with wide resection [4]. In this series, we present findings from three cases of CCOC that presented at our institution over the past six years.

## 2. Materials and Methods

Patient charts from our institution were reviewed from 2014–2019 to reveal three cases of clear cell odontogenic carcinoma. Patient charts were reviewed for medical history and radiological data. The pathology slides were pulled and reviewed by one or more faculty members

### 2.1. Case 1 

**Figure 1:**
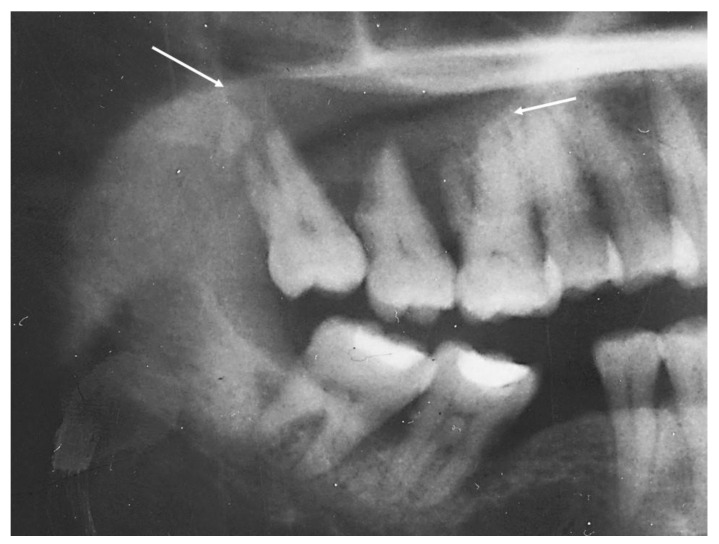
Panoramic X-ray film revealing poorly demarcated radiolucency in the right posterior maxilla.

**Figure 2:**
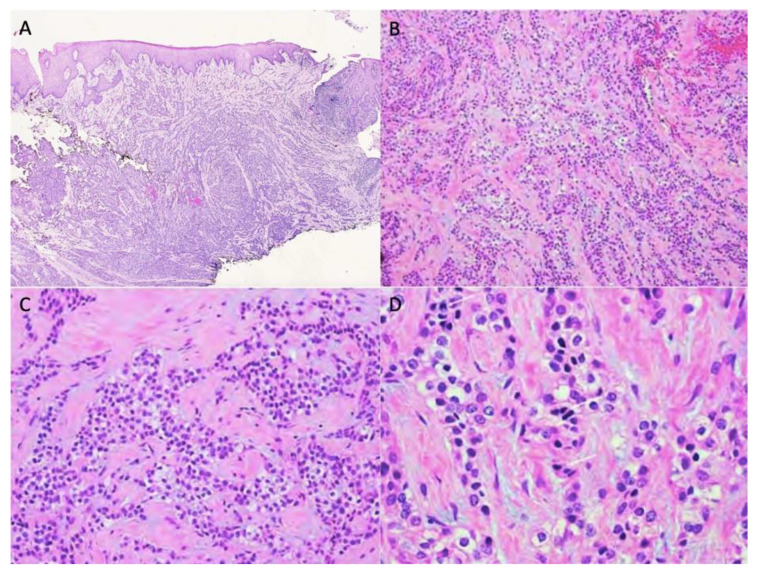
Non-dysplastic parakeratinized surface epithelium with a subepithelial markedly infiltrative, non-encapsulated malignant neoplastic odontogenic epithelial proliferation with a prominent clear cell component (**A**, 40×). Neoplastic cells were arranged in anastomosing trabeculae and nests exhibiting nuclear hyperchromasia and pleomorphism and optically clear cytoplasm (**B**,**C**, 100× & 200×, respectively). Nuclear pleomorphism and cytoplasmic clearing (**D**, 400×).

**Figure 3:**
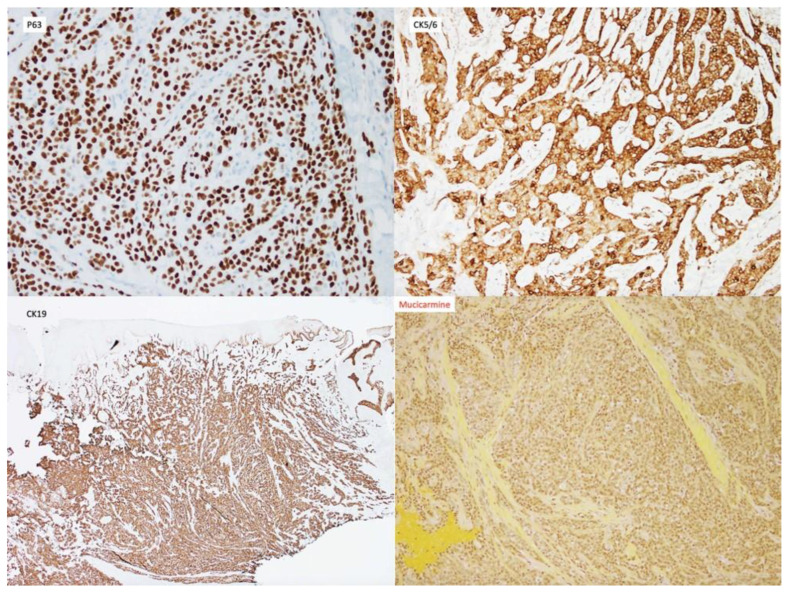
Immunohistochemistry was diffusely positive for p63 (100×), CK5/6 (100×), CK19 (40×) and negative for mucicarmine (100×).

**Figure 4:**
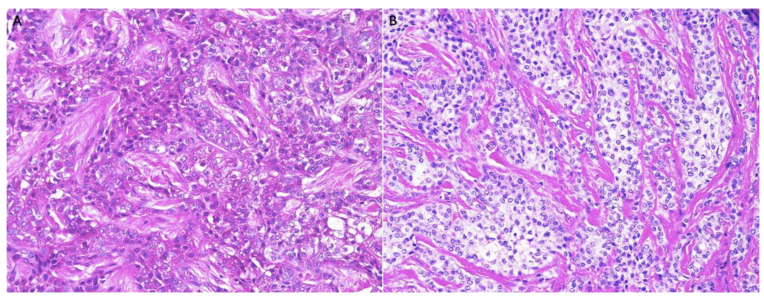
Immunohistochemical staining showing PAS-positive (**A**) and diastase-labile intracytoplasmic glycogen (**B**) (200×).

A 54-year-old woman presented with a 32 × 44 mm poorly demarcated radiolucency in the right posterior maxilla (Figure 1). She reported that the lesion had been present for two years, was tender, and involved the posterior palate. The first and second molars were found to float in the tissue mass. The molars were extracted, and an incisional biopsy was performed. The soft tissue specimen grossly contained multiple tan, firm fragments measuring together 3 × 2 × 1 cm. Histological examination revealed a specimen composed of non-dysplastic parakeratinized oral mucosa supporting a markedly infiltrative, non-encapsulated malignant neoplastic odontogenic epithelial proliferation with a prominent clear cell component, supported by dense fibrous connective tissue stroma (Figure 2A). The neoplastic cells were arranged in anastomosing trabeculae and exhibited nuclear hyperchromasia and pleomorphism surrounded by optically clear, vacuolated cytoplasm (Figure 2B,C). Occasional atypical mitotic figures were observed (Figure 2D). The stroma was hyalinized, densely collagenized, hypocellular, and hypovascularized with lymphocyte aggregates. s. P63, CK5/6, and CK19 were intensely positive in neoplastic areas, while mucicarmine stain was negative (Figure 3). Periodic acid Schiff (PAS) staining showed intracytoplasmic glycogen, which was labile when PAS diastase was used (Figure 4). A diagnosis of clear cell odontogenic carcinoma of the right posterior maxilla was made based on data provided.

### 2.2. Case 2

**Figure 5:**
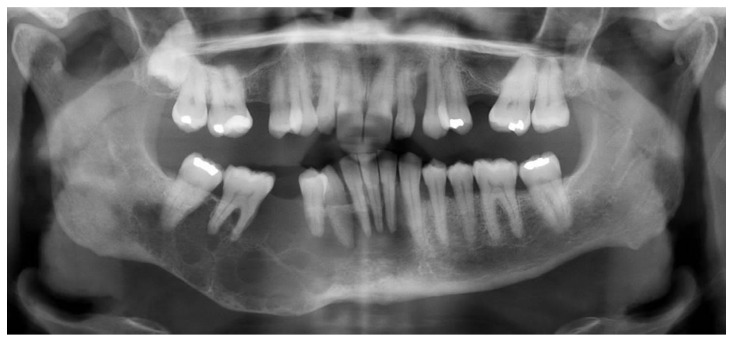
Panoramic X-ray film revealing a 10 cm expansile multilocular radiolucency with poorly demarcated borders in the body of the right mandible.

**Figure 6:**
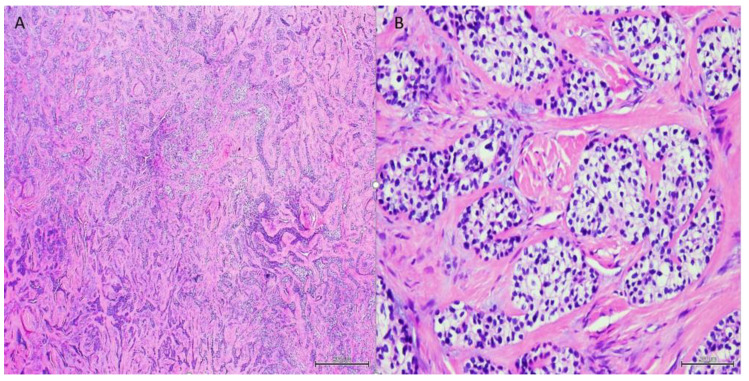
H&E, uncircumscribed neoplastic odontogenic epithelial proliferation, diffusely infiltrating the connect tissue stroma (**A**, 40×). The neoplastic proliferation formed islands of variable size, trabeculae, and nests exhibiting central pleomorphic and hyperchromatic nuclei surrounded by clear cytoplasm (**B**, 200×).

**Figure 7:**
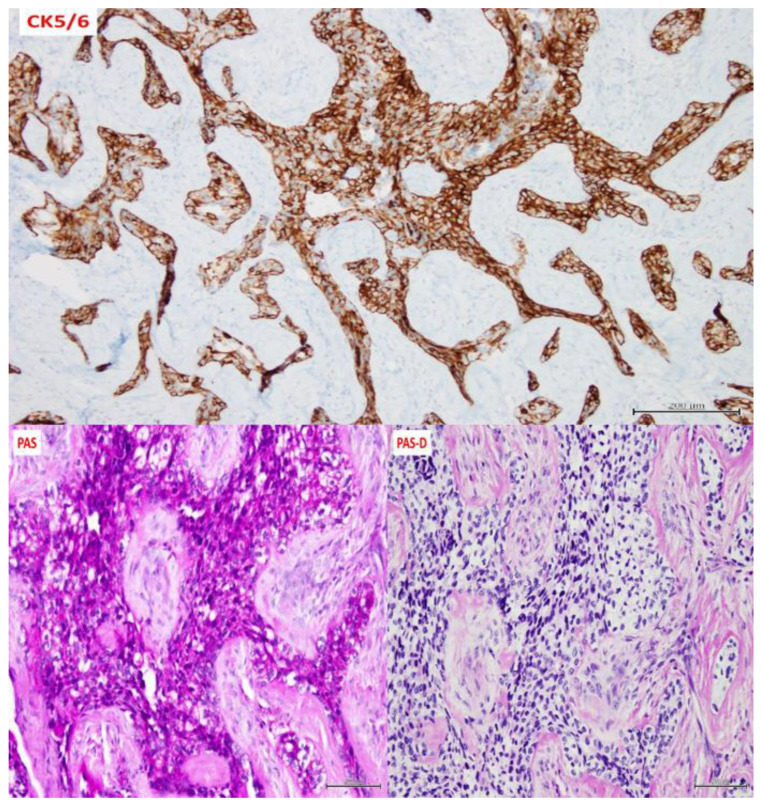
Case 2 immunohistochemistry was diffuse and strongly positive for CK5/6 and PAS- stain which was diastase-labile (200×).

A 49-year-old woman presented with a 10 cm painful expansile multilocular radiolucency in the body of the right mandible for at least 18 months (Figure 5). The most bothersome symptom were pain and numbness in the V3 distribution. An incisional biopsy was performed on the crestal area of the mandibular ridge, and the mass was solid without a cystic component. On gross examination, a 13 × 8 × 7 mm tan and firm soft tissue specimen were recovered. Histologically, the lesional tissue consisted of an uncircumscribed, diffusely infiltrative neoplastic odontogenic proliferation invading the connective tissue stroma (Figure 6A). Neoplastic proliferation formed islands of variable size, trabeculae, and nests. Neoplastic cells exhibited central pleomorphic and hyperchromatic nuclei surrounded by a clear cytoplasm (Figure 6B). Immunohistochemical staining for CK5/6 was diffuse and strongly positive in the neoplastic cells (Figure 7), and PAS staining demonstrated PAS-positive and diastase-labile intracytoplasmic material (Figure 7). The mucicarmine stain was negative. Following the diagnosis, the patient underwent left mandibulectomy, right temporomandibular joint arthroplasty, and mandible reconstruction with left fibula free flap. Additionally, histological sections revealed positive margins that were managed by re-excision and later confirmed with negative margins. Follow-up with examination and imaging revealed no disease recurrence.

### 2.3. Case 3

**Figure 8:**
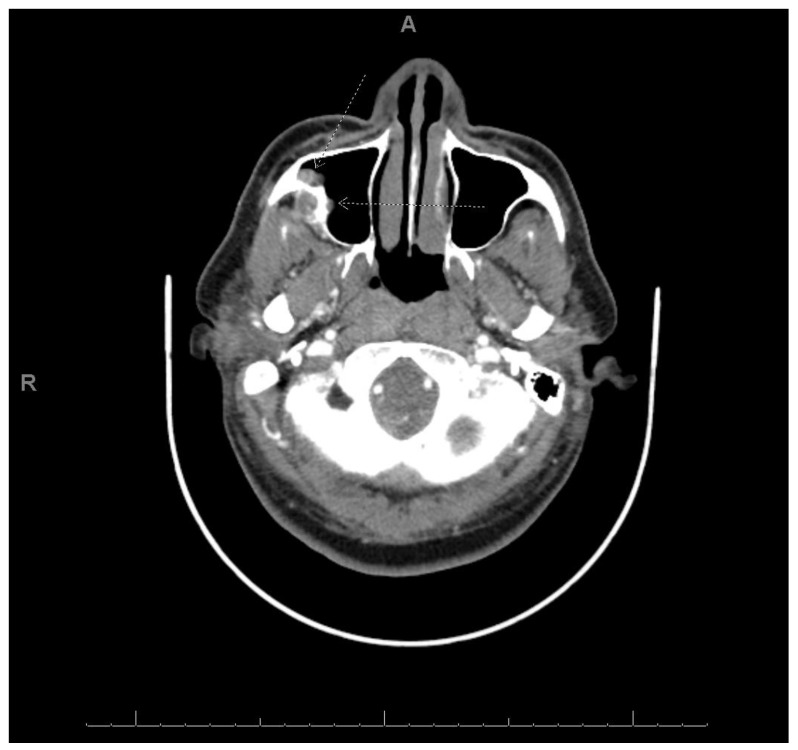
Heterogeneous soft tissue mass involving lateral wall of right maxilla and alveolar process axial view.

**Figure 9:**
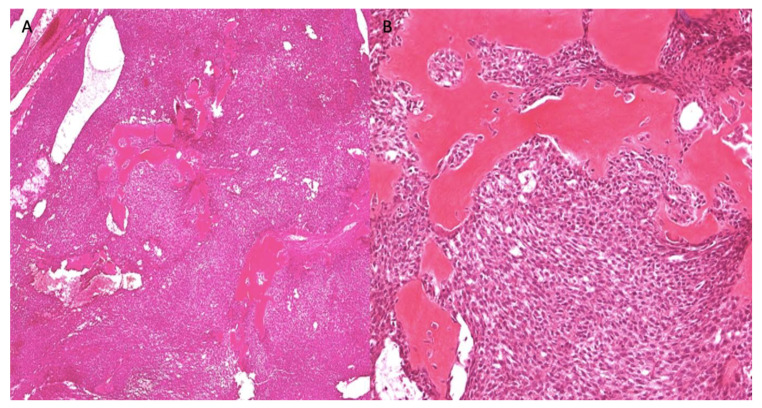
H&E, Infiltrative odontogenic epithelial neoplasm intermixed with eosinophilic dentinoid matrix. Neoplastic proliferation surrounded by reactive cellular osseous trabeculae with osteoblastic rimming (100× (**A**) and 200× (**B**)).

A 40-year-old man was referred by a community dentist to our institution. He reported that he had a mass excised and one of his right upper teeth removed approximately one year prior. The mass at that time was diagnosed as CCOC. Upon presentation to our institution, he complained of a recurrent painless mass located at the same location as the previous mass that had grown over the past several months. A CT scan was performed prior to presentation which revealed a geographic heterogeneous soft tissue mass involving the lateral aspect of the lateral wall of the right maxilla and the alveolar process below the zygomaticomaxillary buttress, measuring 37.5 × 24 × 28.4 mm (Figure 8). An enlarged lower level II lymph node was also noted. Therefore, the patient underwent a right maxillectomy and right neck dissection. Gross examination revealed, a well-circumscribed, 3 × 1.8 × 1.5 cm mass was appreciated. Histological examination revealed an infiltrative odontogenic epithelial neoplasm intermixed with eosinophilic dentinoid matrix. Infiltrative odontogenic epithelial proliferation was present in the form of non-encapsulated sheets, cords, and nests of polygonal cells with central hyperchromatic and slightly pleomorphic nuclei surrounded by clear cytoplasm and focal areas of pale eosinophilic cytoplasm. Occasional mitotic figures are also observed. The neoplastic epithelial sheets and cords were blended with an eosinophilic cellular matrix, without cellular rimming, consistent with the dentinoid deposits. The neoplastic proliferation was surrounded by reactive cellular osseous trabeculae in areas permeated by the neoplastic epithelium (Figure 9). Clear cell odontogenic carcinoma was diagnosed based on hematoxylin and eosin (H&E) staining alone. Following diagnosis, the patient underwent right maxillectomy without orbital exenteration, right selective neck dissection, and forearm free flap reconstruction. Histological sections of the resected mass showed negative margins. The patient then underwent four months of adjuvant radiation.

## 3. Summary of Cases

The cases presented here represent three cases of CCOC that were treated at our institution from 2014 to 2019. Two patients were female, and one was male. The patients were aged between 40 and 55 years. Each patient presented with a mass either in the mandible or within the oral cavity. Two patients presented without symptoms, whereas one patient developed neurological symptoms of pain and numbness in the V3 distribution. Each mass had characteristic radiographic findings of CCOC, consisting of a poorly defined expansive radiolucency. Histological descriptions of the tumors were similar for each case consisting of pleomorphic nuclei surrounded by clear, vacuolated cytoplasm, as well as hyalinized stroma that was densely collagenized, hypocellular, and hypovascular. Immunohistochemical staining was performed in only two cases in which both were positive for CK5/6 and PAS-positive diastase-labile intracytoplasmic glycogen. Of the two cases, one case stained positive for both p63 and CK19. In addition, staining for all three cases was negative for mucicarmine and Congo red, which ruled out clear cell mucoepidermoid carcinoma and clear cell calcifying epithelial odontogenic tumor (CCCEOT), respectively. The diagnosis of one case was made solely on the basis of characteristic dentinoid deposits on H&E staining. See Table 1.

## 4. Discussion

Clear cell odontogenic carcinoma (CCOC) is a rare malignant odontogenic epithelial neoplasm. Although initially classified as a benign neoplasm, CCOC was reclassified as a malignant carcinoma because of its aggressive behavior, with local destruction, frequent recurrence, and metastasis [1]. Most cases commonly occur in patients during their 5th and 6th decades of life, with a male-female-ratio of 1:1.8 [5]. Clinically, CCOC typically presents as asymptomatic swelling that may be accompanied by pain, tooth mobility, and gingival symptoms. On rare occasions, this tumor can cause bleeding, oral mucosal expansion, paresthesia, and oral ulceration [6]. The treatment of choice for CCOC is surgical resection with wide margins due to a higher recurrence rate in those treated conservatively than in those who underwent resection (86.7% vs. 29%) [7]. Given the limited number of reported CCOC cases, there remains a significant need to highlight their unique behavior and diagnostic features.

Distinguishing CCOCs from other jaw malignancies relies largely on their unique histological characteristics and immunohistochemical expression. CCOCs exhibit three distinct histological patterns: monophasic, biphasic, and ameloblastic [8]. The monophasic variant consists entirely of clear cells within nests and cords. The biphasic variant, the most common, consists of two cell populations; clear cells and hyperchromatic polygonal cells with eosinophilic cytoplasm. The ameloblastic variant consists of palisading clear and columnar cells with ameloblast-like differentiation at the periphery of the tumor islands [1,9]. Very rarely, CCOCs produce variable amounts of dentinoid in mature fibrous connective stroma. CCOC with a dentinoid matrix has also been described as “odontogenic carcinoma with dentinoid” (OCD) [10,11,12].

Although clear cells are a hallmark of CCOC, they have also been observed in other tumors, including salivary, renal, thyroid, and prostate tumors. Therefore, immunohistochemistry is often used to confirm CCOC and rule out its differential diagnoses. CCOCs commonly stain positive for cytokeratin, including CK5, CK6, CK8, CK13, CK14, CK18, CK19, CK20, AE1/AE2, p63, and epithelial membrane antigen (EMA). In addition, staining for CCOC is typically negative for S-100, HMB-45, mucicarmine, congo red, desmin, SMA, CD31, CD45, and GFAP [2,13].

Recently, DNA microarray studies have provided a genetic basis for diagnosis with 83.3% of CCOCs exhibiting translocation of the *EWSR1* gene, most commonly with activating transcription factor 1 (*ATF1*) [14]. However, such a rearrangement is also exhibited in clear cell carcinoma of the minor salivary glands, which further necessitates the additional use of immunohistochemistry, together with clinical and radiologic correlations. In addition, genetic analysis of OCD has demonstrated pathogenic mutations in *CTNNB1* and *APC* with persistent Wnt signaling activation [15].

In this series, we report three cases of CCOC and describe the unique histopathological and immunohistochemical findings. One case exhibited unique cytologic features, with the presence of an eosinophilic dentinoid matrix surrounded by reactive cellular osseous trabeculae in areas permeated by the neoplastic epithelium. This CCOC with dentinoid, also referred to as odontogenic carcinoma with dentinoid (OCD), is a rare occurrence. To date, only ten cases have been reported in the literature of which four exhibited recurrence [11]. Dentinoid is frequently observed in several benign odontogenic tumors including odontomas, dentinogenic ghost cell tumors, and adenomatoid odontogenic tumors [10,11]. However, dentinoid-producing odontogenic tumors are very rare and are not recognized in the latest *World Health Organization histological classification of tumors* (2017) [12]. Our patient with dentinoid exhibited recurrence one year after the removal of a previous CCOC.

The differential diagnosis for CCOC includes other tumors that exhibit histopathological features similar to those of clear cells. Such differential diagnoses include clear cell variants of calcifying epithelial odontogenic tumors (CCCEOT, also known as the Pindborg tumor), hyalinizing clear cell carcinoma (CCC), ameloblastic carcinoma, intraosseous clear cell mucoepidermoid carcinoma (CCMEC), acinic cell carcinoma, intraosseous variant of melanoma, and metastatic tumors of renal, thyroid, and prostate origins [2,16,17]. Alternative histopathological features and immunohistochemical staining play a pivotal role in differentiating CCOCs from such diagnoses. Differential diagnosis of odontogenic origin includes clear cell ameloblastoma and CCCEOT. Clear cell ameloblastoma is characterized by peripheral tall columnar cells with palisading and reverse nuclear polarity on histology, and positive expression of calretinin and CK19 on immunohistochemistry. CCCEOT is characterized by calcified concentric rings (Liesegang rings) and amyloid deposits within the stroma that display apple-green birefringence when stained with Congo red and viewed under polarized light [16]. Such Liesegang rings and amyloid deposits were not observed in any of the cases presented herein. Additional distinguishing features exhibited across other differential diagnoses were not observed in these cases making CCOC the most likely diagnosis (Table 2).

Given the aggressive nature of CCOC and the high rate of recurrence, surgical resection with wide clear margins is the gold standard treatment [1,3,4]. All three patients underwent surgical resection with a wide margin and were followed for 12 months without evidence of recurrence.

Owing to the rarity of CCOC and the limited reported cases in the literature, this report of three cases with corresponding histopathological and immunohistochemical profiles provides further insight into the diagnostic and prognostic factors of CCOC. Furthermore, the presence of a dentinoid matrix in CCOC is very rare with only ten cases reported.

## 5. Conclusions

CCOC is a rare malignant odontogenic tumor with an aggressive behavior. Despite its rare occurrence, CCOC should be considered in the differential diagnosis of jaw tumors with clear cell components. Histopathologic and immunohistochemical profiles are critical for distinguishing CCOCs from other differential diagnoses. Resection with a wide margin is the treatment of choice, as conservative management with curettage is associated with high recurrence rates. In addition, long-term follow-up is advised to monitor recurrence or metastasis.

## Figures and Tables

**Table 1:** Case Summaries.

	Case 1	Case 2	Case 3
Age/Sex	54-year-old female	49-year-old female	40-year-old male
Presentation	Painful mass	Painful mass	Mass recurrence
Location	Right maxilla	Right mandible	Right maxilla
Staining/IHC	PAS (+) D-labile Mucicarmine (−), p63 (+) CK5/6 (+), CK19 (+)	PAS (+) D-labile Mucicarmine (−) CK5/6 (+)	H&E only
Morphology	Infiltrative, non-encapsulated malignant neoplastic odontogenic epithelial proliferation with clear cell component in a dense fibrous connective tissue stroma. Neoplastic cells arranged in anastomosing trabeculae. Nuclear hyperchromasia and pleomorphism surrounded by clear, vacuolated cytoplasm. Stroma was hyalinized, densely collagenized, hypocellular, and hypovascular.	Neoplastic odontogenic epithelial proliferation diffusely infiltrated the connective tissue stroma. Neoplastic cells arranged in islands of variable size, trabeculae, and nests. Nuclei were hyperchromatic, central, and pleomorphic surrounded by clear cytoplasm.	Infiltrative odontogenic epithelial neoplasm intermixed with eosinophilic dentinoid matrix. Neoplastic cells arranged in infiltrative, non-encapsulated sheets, cords, and nests of polygonal cells with central hyperchromatic and slightly pleomorphic nuclei surrounded by clear cytoplasm and occasional pale eosinophilic cytoplasm. Occasional mitotic figures were noted. Neoplastic epithelial sheets and cords blended with eosinophilic cellular matrix without cellular rimming consistent with dentinoid deposits.
Procedure	Right maxillectomy with negative margins	Left mandibulectomy Right temporomandibular joint arthroplasty Left fibula free flap	Right maxillectomy without orbital exenteration Right selective neck dissection Forearm free flap Four months adjuvant radiation

PASD = Periodic acid Schiff with diastase, CK = Cytokeratin, H&E = hematoxylin and eosin.

**Table 2:** Differential diagnoses of CCOC.

Differential Diagnosis	Distinguishing Features
Odontogenic origin	
Clear cell ameloblastoma	Palisading clear cells and columnar cells with ameloblast-like differentiation on histology Immunopositive for calretinin, CK8, CK13, CK19
Clear cell calcifying epithelial odontogenic tumor	Liesgang’s calcifications and amyloid deposits on histology with apple-green birefringence on Congo red stain
Salivary origin	
Mucoepidermoid carcinoma	Pale basophilic foamy cytoplasm on histology Positive alcian blue stain, mucicarmine, and mucin Immunopositive for CK19
Myoepithelial carcinoma	Immunopositive for S100, alpha SMA, calponin, vimentin
Acinic cell carcinoma	Interspersed basophilic granules on histology Positive for PTAH stain Immunopositive for S100, pancytokeratin, vimentin, CEA, GFAP
Hyalinizing clear cell carcinoma	Peripheral palisading and hyalinization of stroma on histology Immunopositive for pancytokeratin and EMA
Metastatic origin	
Renal cell carcinoma	Immunopositive for CD10 and PAX8
Thyroid carcinoma	Immunopositive for TTF-1, Thyroglobulin
Prostatic carcinoma	Increased serum PSA
Melanotic origin	
Amelanotic melanoma	Immunopositive for Masson-Fontana stain, melan A, and HMB-45

CK = cytokeratin, alpha SMA = alpha smooth muscle actin, PTAH = phosphotungstenic acid hematoxylin, CEA = carcinoembryonic antigen, GFAP = glial fibrillary acidic protein, PAX8 = Paired-box gene 8, EMA = epithelial membrane antigen, CD = cluster of differentiation, PSA = prostate specific antigen, HMB-45 = homatropine methyl bromide.

## Data Availability

Not applicable.
